# Regional Variability in Sugar and Amino Acid Content of U.S. Soybeans and the Impact of Autoclaving on Reducing Sugars and Free Lysine

**DOI:** 10.3390/foods13121884

**Published:** 2024-06-15

**Authors:** Takehiro Murai, Seth Naeve, George A. Annor

**Affiliations:** 1 Department of Food Science and Nutrition, College of Food, Agricultural and Natural Resource Sciences, University of Minnesota, 1334 Eckles Avenue, St. Paul, MN 55108, USA; murai021@umn.edu; 2 Department of Agronomy and Plant Genetics, College of Food, Agricultural and Natural Resource Sciences, University of Minnesota, 411 Borlaug Hall 1991 Upper Buford Circle, St. Paul, MN 55108, USA; naeve002@umn.edu

**Keywords:** soybean, sucrose, glucose, lysine, Maillard

## Abstract

Exploring the sugar and amino acid content variability and the influence of thermal processing on these in soybeans can help optimize their utilization in animal feed. This study examined 209 samples harvested in 2020 and 55 samples harvested in 2021 from across the U.S. to assess their sugar variability and amino acid variability. Harvest regions included the East Corn Belt, West Corn Belt, Mid-South, East Coast, and the Southeast of the U.S. In addition to the sugar and amino acid contents, protein, oil, and seed size were also analyzed. Samples from 2021 were evaluated for their sugar and amino acid contents before and after autoclaving the seeds at 105–110 °C for 15 min. For the samples harvested in 2020, sucrose (4.45 g 100 g^−1^) and stachyose (1.34 g 100 g^−1^) were the most prevalent sugars. For the samples harvested in 2021, L-arginine (9.82 g 100 g^−1^), leucine (5.29 g 100 g^−1^), and glutamate (4.90 g 100 g^−1^) were the most prevalent amino acids. Heat treatment resulted in an 8.47%, 20.88%, 11.18%, and 1.46% median loss of free lysine, sucrose, glucose, and fructose. This study’s insights into the variability in sugar and amino acid content and the heat-induced changes in the nutritional composition of soybeans provide a reference for improving soybean quality assessment and optimizing its use in animal feed formulations in the U.S.

## 1. Introduction

Soybean (Glycine max L. Merr.) is a widely cultivated crop known for its high nutritional value, consisting of approximately 35% protein, 17% oil, and 31% carbohydrate [1]. A significant portion of the soluble carbohydrates in soybeans consists of sucrose, stachyose, and raffinose [2]. While sucrose is a desirable sugar for animal feed due to its high digestibility and palatability [3], stachyose and raffinose are often undesired due to their hindrance of animal growth [4,5]. Variability in sugar contents in different soybean varieties and from different locations have been reported [6,7,8]. Hou and others [6] analyzed 241 soybeans from the worldwide germplasm collection which were grown in Costa Rica and identified new classes of soybeans containing high concentrations of glucose and fructose. Jiang and others [7] investigated the variability in individual sugars in soybeans across 323 germplasms grown in three locations within South Dakota, where they reported significant genotypic variation for all individual sugars. Additionally, they observed genotype–environment interactions for sucrose and raffinose across the three different environmental conditions. Kumar and others [8] reported a higher sucrose content in soybeans grown in cooler locations in India but did not address the variability in glucose and fructose among the varying locations. While these studies have reported on the sugar contents in different soybean varieties, to our knowledge, no study has investigated the difference in sugar content of soybeans grown in different states within the U.S.

Just as some sugars in soybeans are more desirable than others, certain amino acids are desired more than others [9]. For instance, lysine stands out as an important amino acid in swine nutrition, serving as an essential building block for proteins and peptides [10]. Variability in amino acid composition of soybean meals within the U.S. has been explored by Karr-Lilienthal and others [11], where samples were collected from 55 soybean processing plants located over the seven maturity zones, as described by Grieshop and others [12]. However, as the study by De Borja Reis and others [13] has shown, the amino acid composition of soybeans in the U.S. is subject to changes in breeding aims and practices. Understanding the current variability in the amino acid composition of U.S. soybean meal can help monitor the changes for future surveys to come.

Soybeans are typically not used directly as animal feed in their raw seed form; instead, they are processed into soybean meal as a by-product of soybean oil production, involving various heat treatments [14]. Once dried to a moisture content of around 13%, the seeds are cracked using rolls to facilitate dehulling and flaking. Removing the hulls leads to the production of a high-protein soybean meal, enhancing its nutritional value per unit weight. The conditioned seeds are then flaked and subjected to solvent extraction to separate the soybean meal from soybean oil. The soybean meals are then heat treated at temperatures ranging from 100 to 105 °C for 15 to 30 min to eliminate any residual organic solvent. Subsequently, the soybean meal undergoes additional drying processes to adjust its moisture content before grinding. While these heat treatments are advantageous in deactivating anti-nutritional factors [15], there is a concern about the reduction in amino acid content due to the Maillard reaction. Lysine is particularly susceptible to the Maillard reaction due to its ε-amino group and becomes less bio-accessible by reacting with reducing sugars such as glucose [16,17]. Many studies on the Maillard reaction in thermally treated soybeans have successfully quantified Maillard reaction products [18,19]. In addition, the work of Parsons and others [20] sheds light on the impact of different autoclaving conditions on lysine loss in soybeans. However, their study was limited to two varieties of soybean. 

This study is composed of three objectives. The first objective is focused on investigating the variability in sugars harvested among different states in the U.S. The second objective investigates the variability in amino acids among different states in the U.S. The third objective investigates the impact of thermal processing on the loss of lysine and reducing sugars in soybean samples harvested across the U.S. 

## 2. Materials and Methods

### 2.1. Plant Material

Samples for this study were obtained through the U.S. Soybean Quality Survey conducted by the University of Minnesota. Sample kits were sent out to 5800 farmers across the U.S. each year in the years 2020 and 2021, where the sampling size for each state was based on the amount of soybean production. A total of 1586 samples were returned in 2020 and 1484 in 2021. Samples were then analyzed for protein and oil content by Near-Infrared Spectroscopy using a PerkinElmer diode array instrument (PerkinElmer, Waltham, WA, USA). A subset of samples was selected from each set to represent a range of protein and oil content for use in this study. 

A total of 209 soybean cultivars from the 2020 harvest year were selected for sugar analysis in this study. Samples were harvested from the Eastern (55) (ECB) and Western Corn Belts (122) (WCB), Mid-South (30) (MDS), and East Coast (2) (EC) regions. For the analysis of amino acid components, 55 soybean cultivars from the 2021 harvest year were selected. The samples for the amino acid analysis were obtained from the ECB (11) WCB (32), MDS (9), EC (1), and Southeast (2) (SE) regions. A smaller set of samples was identified from 2021 due to resource limitations related to additional analyses of the 2021 set. Beyond sampling, to provide a range of composition based on protein and oil content and to maintain representation of U.S. soybean production by region, sampling was random. The 2020 samples represented 175 unique varieties, and only 18 varieties were represented by more than one sample. The 2021 samples represented 54 varieties and 1 variety was represented by more than one sample. The varietal distribution of the smaller sets well represents the distribution of samples across many varieties found in the full sample sets.

### 2.2. Sample Preparation

The sample material was kept in a temperature-controlled cold room at 4 °C until further analysis. For the sugar analysis, 200 g of soybean seeds of each cultivar was ground using a GRINDO MIX GM 200 laboratory knife mill (F. Kurt Retsch GmbH and Co. KG, Haan, Germany) at a speed of 10,000 rpm for 2 min. For the total and free amino acid analysis, 50 g of seeds from each cultivar was ground using a coffee grinder (Sunbeam Products, Inc., Boca Raton, FL, USA) for 5 min in the fine-grind mode. The flour samples were then defatted via the Soxhlet method with petroleum ether for a duration of 4 h. Defatted samples were then autoclaved at between 105 and 110 °C for 15 min and dried at 50 °C in a convection oven for 16 h to observe the effects of thermal processing on the amino acid compositions.

### 2.3. Analysis of Sugar Component

Samples from 2020 were analyzed for their sugar component with a Dionex 5000+ high-performance anion-exchange chromatographic system coupled with pulsed amperometric detection (HPAEC-PAD) (Thermo Fisher Scientific Inc., Waltham, MA, USA), following the method reported by Giannoccaro and others [21] with slight modifications. A total of 1 g of soybean flour was vortexed in a centrifuge tube with a water-to-sample ratio of 5:1 (*v*/*w*) at 25 °C. The solution was allowed to settle at room temperature for 15 min, followed by centrifugation at 15,000 rpm for 10 min until solid particles had formed at the tube’s bottom. A total of 2 ml of the supernatant was transferred to another centrifuge tube, and 3 mL of acetonitrile was added to aggregate the protein. The tube was shaken and left to rest at room temperature for 30 min before centrifugation at 1500× *g* for 10 min. A total of 1 ml of the supernatant was transferred to a glass test tube and dried on a heating block at 80 C for approximately 1 h. The remaining sample residue was re-dissolved, transferred to a 100 mL volumetric flask, and made up to volume with a 90 mM NaOH solution. The final solution was filtered into a 2 mL glass vial using a 0.45 um membrane filter. The sample solution was injected into the HPAEC-PAD through a 25 μL injection loop and eluted with a 90 mM NaOH solution through the CarboPac PA10 anion-exchange resin column (250 × 4 mm) along with its corresponding guard column (50 × 4 mm). Standard curves were generated using sugar standards purchased from Sigma-Aldrich (St. Louis, MO, USA) to calibrate and quantify the eight different sugars. The analyzed sugars include arabinose, xylose, glucose, fructose, sucrose, melibiose, stachyose, and raffinose.

### 2.4. Analysis of Total Amino Acids

The protocols outlined in the Product Manual for AAA-Direct, Dionex Amino Acid Analyzer (Thermo Fisher Scientific Inc., Waltham, MA, USA), were followed with a slight modification for the preparation of protein hydrolysate samples. Approximately 2–6 mg of flour samples was hydrolyzed at 110 °C under vacuum conditions using 6 M HCl for a period of 16 h. Subsequently, the samples were neutralized with 6 M NaOH. Amino acids were separated with the AminoPac™ PA10 column (2 × 250 mm) along with its corresponding guard column (2 × 50 mm). The eluents, eluent gradient program, and conditions used followed the specifications outlined in Application note 163 titled “Determination of Protein Concentrations Using AAA-Direct™” (Thermo Fisher Scientific Inc., Waltham, MA, USA) [22] (Table 1). External standard curves were generated using a commercial amino acid standard mix AAS18 (Millipore Sigma, Burlington, MA, USA). The results obtained from this method include 17 amino acids, namely arginine, lysine, alanine, threonine, glycine, valine, serine, proline, isoleucine, leucine, methionine, histidine, phenylalanine, glutamate, aspartate, cysteine, and tyrosine. Tryptophan is not included in the results obtained from this protocol as it does not survive the hydrolysis process.

### 2.5. Analysis of Free Amino Acid and Sugars

Sample solutions for the free lysine and sugars were prepared by mixing 2–6 mg of defatted soy flour in double-distilled water and allowing the mixture to rest at room temperature for 30 min. The mixture was then centrifuged at 13,000 rpm for 5 min and the supernatant was filtered into a 2 mL glass vial using a 0.45 um membrane filter. The eluents, eluent gradient program, and conditions used were as described in Section 2.3.

### 2.6. Analysis of Protein Content

Protein content was determined by following the protocols of the AOCS Official Method Ba 4e-93 [23].

### 2.7. Analysis of Oil Content

Oil content was determined by following the protocols of the AOCS Official Method Ac 3–44 [24].

### 2.8. Statistical Analysis

All chemical analyses were performed in duplicates. A one-way analysis of variance (ANOVA), Tukey–Kramer honest significant difference (HSD) test at a 95% confidence level, and Pearson correlation were performed with R 4.3.1 (R Development Core Team). Box plots and choropleth maps were generated with Python 3.10.12 (Python Software Foundation). Sample codes for the ANOVA analysis, Tukey–Kramer HSD test, Pearson correlation, and data visualization can be found at https://github.com/murai021/soybean_survery_2020_2021.git (accessed on 12 June 2024).

## 3. Results and Discussion

### 3.1. Sugar Composition of Soybeans

The sugar composition of the soybean samples is summarized in Table 2. Significant differences (*p* < 0.05) among states were observed for glucose, fructose, sucrose, raffinose, arabinose, xylose, and melibiose (Table 3). Significant differences (*p* < 0.05) among regions were observed for glucose, fructose, sucrose, raffinose, arabinose, and melibiose (Table 4). The average glucose, fructose, sucrose, raffinose, stachyose, arabinose, xylose, and melibiose contents were 0.43 g, 0.21 g, 4.45 g, 0.34 g, 1.34 g, 0.04 g, 0.01 g, and 0.02 g per 100 g of soybean, respectively. Sucrose and stachyose were the most abundant sugars, which aligns with findings from past studies [6,25]. Sucrose content was lowest in samples from Louisiana (2.43 g) and highest in samples from Wisconsin (5.51 g). The average sucrose content observed in this study falls within the range of the average sucrose content of U.S. soybeans reported in the past by Hou and others [6], which was 4.14 g. However, the average stachyose content in this study (1.34 g) was lower than the U.S. average (2.99 g) and the global average (3.17 g) reported in the same study. Differences between the observed and reported values may be due to differences in the germplasm and growing environment. All samples from the study by Hou and others [6] were grown in Costa Rice, while the samples in this study were grown in various states and regions within the U.S. Stachyose was found to have no significant differences among the states (Table 3) (*p* > 0.05). There were also no significant differences in xylose and stachyose content among the regions (Table 4) (*p* > 0.05).

**Figure 1:**
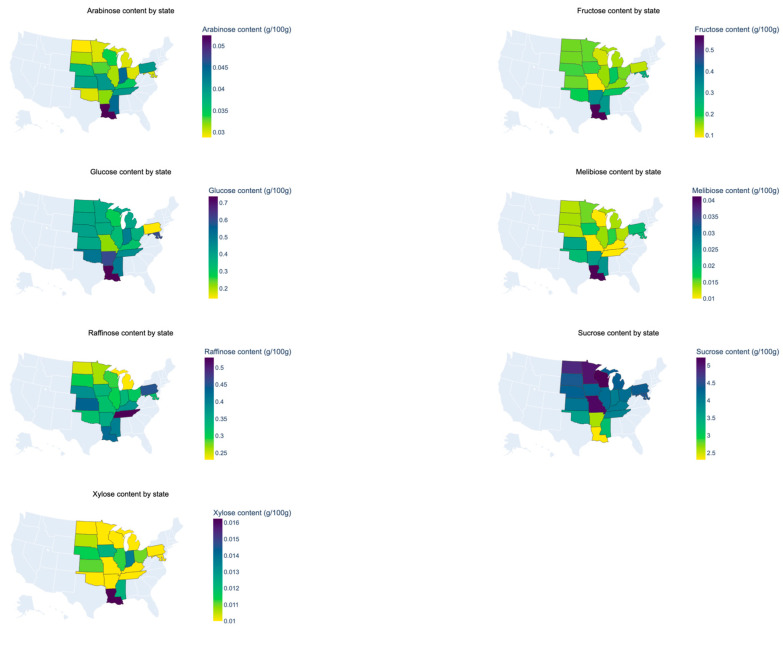
Choropleth maps of sugars of soybeans. Samples were from 2020.

Samples from the Mid-South contained the highest content of glucose (0.60 g 100 g^−1^), fructose (0.39 g 100 g^−1^), raffinose (0.42 g 100 g^−1^), melibiose (0.03 g 100 g^−1^), and arabinose (0.05 g 100 g^−1^), while samples from the West Corn Belt had the highest sucrose content (4.77 g 100 g^−1^) (Table 3). Samples from the East Corn Belt and East Coast had the lowest glucose content (0.39 g 100 g^−1^), the East Corn Belt had the lowest fructose content (0.17 g 100 g^−1^), and the Mid-South region had the lowest sucrose (3.12 g 100 g^−1^) content (Table 4). This pattern of higher sucrose concentrations in northern regions and higher glucose and fructose contents in southern regions was consistently observed at the state level. As visually represented in Figure 1, Wisconsin had the highest sucrose content (5.51 g 100 g^−1^), while Louisiana had the lowest (2.43 g 100 g^−1^). In contrast, Louisiana also had the highest glucose (0.78 g 100 g^−1^) and fructose (0.60 g 100 g^−1^) content, while states from the northern regions such as Minnesota (0.40 g, 0.18 g 100 g^−1^) and Wisconsin (0.30 g, 0.13 g 100 g^−1^) had lower glucose and fructose contents. Past research has also shown sucrose content to increase in cooler regions [8,26]. In terms of glucose and fructose, while past studies have shown differences in these monosaccharide contents among different soybean varieties [6], no study has specifically compared soybeans grown and harvested in different regions specifically in the U.S. according to our knowledge.

The observed differences in sugar content may be explained by the biological responses of soybean plants to various temperature conditions. During the day, soybeans engage in photosynthesis, a process needed for their growth, while at night, they primarily consume the sugars accumulated throughout the day [27]. Research by Yang and others [28] found elongated high night temperatures to decrease sucrose accumulation in soybean seeds and increase accumulation in soybean leaves. Samples from their study exposed to high night temperatures also showed decreased expressions of sucrose synthase and sucrose phosphatase. Other studies also reported high night temperatures to increase carbohydrate usage in plants [29], which may explain the increased glucose levels as glucose serves as a direct energy source in plant growth compared to sucrose.

### 3.2. Total Amino Acid Composition of Soybeans

The results of the total amino acid analysis are summarized in Table 2. Arginine was the most abundant amino acid (9.82 g 100 g^−1^), followed by leucine (5.29 g 100 g^−1^) and glutamate (4.90 g 100 g^−1^). Research conducted by Karr-Lilienthal and others [11] found similar levels of leucine (4.09–4.30 g) but reported lower arginine (3.77–4.05 g) and higher glutamate (9.39–10.15 g) contents. Moreover, a study by De Borja Reis and others [13], which analyzed amino acids in soybean genotypes released between 1980 and 2014 in the U.S., indicated that while leucine content was not influenced by genotype, both arginine and glutamate were. These finding aligns with the comparisons observed in our analysis.

No significant differences in total amino acid compositions were observed among states and among regions in this study. There are mixed reports on the variability in total amino acids among harvest regions. For example, research by Wolf and others [26] showed amino acid composition to not be affected by growing temperatures, while research by Assefa and others [30] showed a negative relationship between amino acid concentration and latitude. Differences in results could be due to the range of soybean varieties investigated in each study; this study investigated 55 samples from 19 U.S. states, Wolf [26] investigated the effect of temperature on one singular variety, and Assefa [30] investigated over 30,000 data points of soybeans collected from 14 U.S. states.

### 3.3. Protein Content of Soybeans

The average protein content for samples obtained in 2020 was 38.91 g, while the average for samples obtained in 2021 was 39.60 g (Supplementary Table S1) per 100 g of seeds. Protein content was significantly different among the states and among the regions in the samples from 2020, where Kansas had the highest value of 41.30 g among the states and the Mid-South had the highest value of 40.24 g among the regions. Protein content was not significantly different among states but was significantly different among regions in the samples from 2021, where the Southeast had the highest protein content of 43.83 g and the Mid-South had the second highest protein content with 42.93 g. Protein content is known to increase with higher growing temperatures [26], which aligns with the observations found in this study. Samples from 2021 in general had a higher protein content than samples from 2020. Differences between samples from 2020 and 2021 may be due to the different growing conditions of each year.

### 3.4. Oil Content of Soybeans

The average oil content of samples from 2020 was 22.60 g, while the average of samples from 2021 was 21.90 g (Supplementary Table S1) per 100 g of seeds. Oil content was significantly different among the states and among the regions in the samples from 2020, where Oklahoma had the highest value of 24.64 g. Oil content was not significantly different among the states but was significantly different among the regions in the samples from 2021, where the Southeast had the highest value of 24.11 g. The discrepancy between the two sample sets could be due to the difference in variety and growing conditions in each year. The results from this study contradict the results from a past study, where a negative correlation between latitude and oil content was reported [30]. No regional correlation was observed in this study, which could be due to differences in sampled varieties and sample size.

### 3.5. Seed Size of Soybeans

The average seed size of samples from 2020 was 15.87 g per 100 seeds, while the average seed size of samples from 2021 was 16.28 g per 100 seeds. The West Corn Belt had the largest seed size (15.31 g 100 seeds^−1^) in samples from 2020, while the Southeast had the largest seed size (13.50 g 100 seeds^−1^) in samples from 2021. Seed size was significantly different across states and across regions in the samples from 2021 but not in the samples from 2020 (Table 4, Supplementary Table S2). Research by Yang and others [28] showed seed size to decrease with elevated night temperature, but no correlations of regional latitude with seed size were observed for this study.

### 3.6. Effect of Thermal Processing on Lysine and Sugar Content of Soybeans

**Figure 2:**
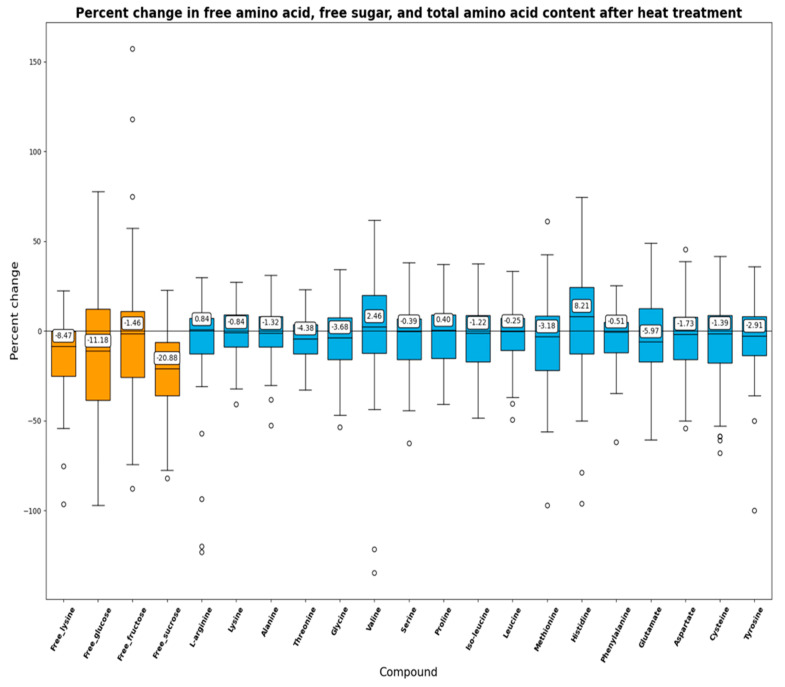
Percent change in free lysine, free sugar, and total amino acid content after heat treatment. Values displayed are median values. Samples are from year 2021 and defatted.

The average free lysine, glucose, fructose, and sucrose content of the soybean meals before autoclaving was 0.03 g, 1.92 g, 1.86 g, and 8.33 g per 100 g, respectively (Table 2). The effects of heat treatment on the loss of free sugars, free amino acids, and total amino acids are plotted as a box plot in Figure 2. Sucrose had the largest fractional decrease in content among the measured sugars, with a median loss of 20.88%, glucose had a loss of 11.18%, and fructose had a loss of 1.46%. Soybeans, just like any other plants, contain invertase, a key enzyme in breaking down sucrose into glucose and fructose for the plant’s energy resource and signaling molecules [31,32]. Invertase is most active at temperatures between 40 and 60 °C, which could explain the decrease in sucrose content [33]. Therefore, the observed changes in glucose and fructose levels cannot be directly attributed to the formation of Maillard products, as they may also result from the enzymatic breakdown of sucrose.

Total lysine had a median loss of 0.84%, while free lysine experienced a more substantial median loss of 8.47%. The loss of lysine observed in this study was less than that reported by Parsons and others [20], which was 15% and 22% upon autoclaving for 40 and 60 min at 121 °C. While the temperatures employed (105–110 °C vs. 121 °C) were different in the study by Parson and others [20], the combined results suggest a positive correlation between lysine loss and the intensity of heat treatment.

### 3.7. Correlation between Traits of Soybeans

**Figure 3:**
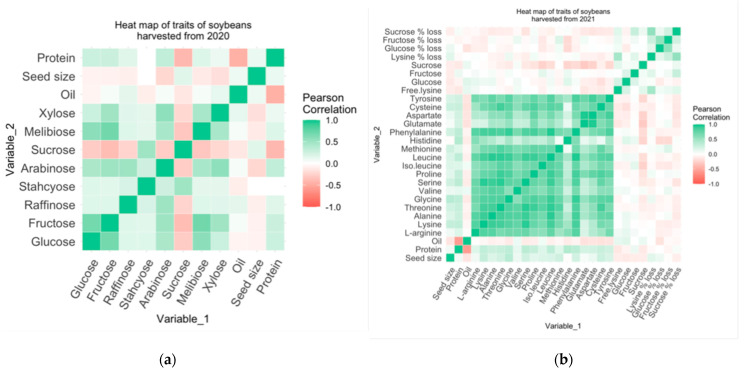
Heat maps of soybean trait correlations. Actual Pearson correlation values can be found in Supplementary Tables S3 and S4. (**a**) Heat map of traits of soybeans harvested in 2020; (**b**) heat map of traits of soybeans harvested in 2021.

Figure 3 displays the heat maps generated to represent the correlations among soybean traits. Sucrose was inversely correlated to all the sugars except for stachyose, which is contradictory to the research by Hou and others [6] and Hymowitz and Collins [34]. Soybeans from the world germplasm studied by Hou and others [6], grown in Costa Rica, showed a negative correlation (−0.68) between sucrose and stachyose. Hymowitz and Collins [35] also report a negative correlation (−0.29) between sucrose and stachyose among the 60 germplasms collected from the U.S. Regional Soybean Laboratory (Urbana, IL). On the other hand, Hartwig and others [35] report a low positive correlation (0.01) between sucrose and stachyose among the 40 germplasms grown in Stoneville, Missouri. Glucose and fructose were positively correlated with each other in both sample sets from 2020 (0.69) and 2021 (0.14), which generally aligns with the correlation (0.99) reported by Hou and others [6].

Protein and oil content were found to be inversely correlated in both sets of samples from 2020 (−0.46) and 2021 (−0.58), which has been observed in past studies [36]. Free lysine loss had a mild positive correlation with free lysine (0.46), glucose (0.03), and fructose (0.16) content. Fructose content had a higher correlation with lysine loss than glucose content, which can be attributed to fructose molecules being more active in initial stages of the Maillard reaction than glucose [37]. Seed size was positively correlated with protein and oil content, which was as expected since oil and protein make up nearly 60% of the total storage of soybean seed [38]. Similarly, sucrose was found to be positively correlated with seed size. This correlation is consistent with past research showing that high night temperatures decrease both sucrose content and seed size, suggesting a link between these variables [28].

### 3.8. Limitations

Some limitations exist in this study. The aim of the sampling methodology was to grasp the nationwide variability. Thus, the sample sizes of the harvest regions and states were not uniformly distributed across each group, potentially leading to an inadequate representation of intra-state variability. The observed variation in soybean composition could also be due to inherent genetic variability among cultivars or management practices employed by the soybean producers, as opposed to purely geographical differences. The effects and results of the thermal processing are strictly bound to the specific heating conditions employed in this study and may not be generalizable to different conditions. Often, soybean meals are exposed to multiple thermal processing steps, including thermal processing for removing organic solvents and extrusion processing, whereas only a simple drying process was employed in this study.

## 4. Conclusions

In this study, the composition of soybeans harvested in 2020 and 2021 was investigated for regional variability, along with the effects of thermal processing on the amino acid composition. Variability among states was observed for glucose, fructose, sucrose, raffinose, arabinose, xylose, and melibiose. Variability among regions was observed for glucose, fructose, sucrose, raffinose, arabinose, and melibiose. Sucrose content was higher in northern states, while there were no regional differences in stachyose content. Glucose and fructose contents were found to be higher in southern states. While the average sucrose content of U.S. soybeans has remained similar to the content reported in previous studies, stachyose content appears to have decreased. Protein content was significantly different among the harvest regions, with higher protein contents in the southern region, but no significant differences among states and regions were observed for amino acids. During heat treatment, glucose had a larger decrease compared to fructose, and free lysine experienced a larger decrease by ratio compared to the total lysine content. Understanding these variabilities and the complexities of the Maillard reaction will be valuable for future research and agricultural practices, aiming to optimize soybean quality for various applications.

## Figures and Tables

**Table 1:** Gradient conditions for protein hydrolysates from AAA-Direct™ manual.

Time (Min)	250 mM NaOH (%)	1 M C_2_H_3_NaO_2_ (%)	H_2_O (%)	Curve
Initial	76	24	0	
0.0	76	24	0	
2.0	76	24	0	
8.0	64	36	0	8
11.0	64	36	0	
18.0	40	20	40	8
21.0	4	16	40	5
23.0	14	16	70	8
42.0	14	16	70	
42.1	20	80	0	5
44.1	20	80	0	
44.2	76	24	0	5
75.0	76	24	0	

**Table 2:** Average sugar and amino acid composition of soybean seeds and soybean meal.

Parameter	Average	Parameter	Average	Parameter	Average
Glucose ^a^	0.43 ± 0.22	Alanine ^b^	2.74 ± 0.88	Phenylalanine ^b^	2.64 ± 0.76
Fructose ^a^	0.21 ± 0.17	Threonine ^b^	2.71 ± 0.75	Glutamate ^b^	4.90 ± 1.65
Sucrose ^a^	4.45 ± 0.96	Glycine ^b^	2.29 ± 0.71	Aspartate ^b^	4.27 ± 1.36
Raffinose ^a^	0.34 ± 0.10	Valine ^b^	3.49 ± 1.22	Cysteine ^b^	0.90 ± 0.27
Stachyose ^a^	1.34 ± 0.19	Serine ^b^	3.81 ± 1.14	Tyrosine ^b^	1.71 ± 0.56
Arabinose ^a^	0.04 ± 0.01	Proline ^b^	4.61 ± 1.39	Free lysine ^b^	0.03 ± 0.04
Xylose ^a^	0.01 ± 0.00	Iso-leucine ^b^	3.83 ± 1.16	Free glucose ^b^	1.92 ± 2.27
Melibiose ^a^	0.02 ± 0.01	Leucine ^b^	5.29 ± 1.54	Free fructose ^b^	1.86 ± 3.24
L-arginine ^b^	9.82 ± 3.31	Methionine ^b^	1.55 ± 0.47	Free sucrose ^b^	8.33 ± 2.14
Lysine ^b^	4.75 ± 1.83	Histidine ^b^	2.29 1.32		

Average values are followed with ± standard deviation. Parameters denoted with “a” are based on samples from year 2020. Parameters denoted with “b” are based on samples from year 2021, defatted, but not autoclaved.

**Table 3:** Differences in traits of soybeans by state.

State	Region	*n*	Glucose	Fructose	Sucrose	Raffinose	Stachyose	Arabinose	Xylose	Melibiose	Oil	Protein	Seed Size
			(g 100 g^−1^)										(g 100 seeds ^−1^)
AR	MDS	5	0.64 ab	0.35 ab	2.75 de	0.37 abcdef	1.19	0.03 abd	0.01 ab	0.02 ab	23.19 abc	40.74 abc	15.86
IA	WCB	35	0.40 a	0.19 a	4.52 ab	0.35 abcde	1.35	0.04 ab	0.01 ab	0.02 a	23.49 a	38.09 a	15.29
IL	ECB	24	0.35 a	0.16 a	4.42 a	0.31 acf	1.3	0.03 a	0.01 ab	0.01 a	23.11 ab	37.64 a	15.58
IN	ECB	11	0.52 ab	0.23 a	4.21 ac	0.35 abcdef	1.39	0.05 bcd	0.01 ab	0.02 a	22.31 abc	40.76 bc	15.00
KS	WCB	9	0.41 a	0.18 a	4.35 abc	0.46 b	1.38	0.04 abcd	0.01 ab	0.02 ab	21.73 abc	41.30 abc	15.85
KY	MDS	2	0.33 ab	0.16 ab	4.26 abcde	0.4 abcdef	1.38	0.04 abcd	0.01 ab	0.01 ab	21.73 abc	39.36 abc	14.31
LA	MDS	8	0.78 b	0.60 b	2.43 d	0.45 bd	1.25	0.06 c	0.02 a	0.04 b	23.30 abc	41.14 b	15.85
MD	EC	1	0.63 ab	0.29 ab	4.68 abcde	0.34 abcdef	1.37	0.04 abcd	0.01 ab	0.02 ab	21.45 abc	39.99 abc	16.80
MI	ECB	3	0.41 ab	0.15 a	4.60 abce	0.24 acef	1.22	0.03 abd	0.01 ab	0.02 ab	22.27 abc	40.97 abc	18.87
MN	WCB	16	0.40 a	0.18 a	5.29 b	0.28 cf	1.39	0.03 a	0.01 ab	0.02 a	21.9 bc	38.93 abc	17.30
MO	WCB	1	0.24 ab	0.10 ab	5.39 abce	0.34 abcdef	1.41	0.05 abcd	0.01 ab	0.01 ab	21.9 abc	36.66 abc	18.20
MS	MS	13	0.52 ab	0.33 a	3.38 cde	0.42 bde	1.35	0.05 cd	0.01 ab	0.03 ab	23.30 ab	39.88 abc	16.55
ND	WCB	25	0.39 a	0.17 a	5.12 ab	0.26 f	1.36	0.03 a	0.01 ab	0.01 a	21.35 c	38.24 ac	14.47
NE	WCB	14	0.43 a	0.19 a	4.58 ab	0.4 abde	1.29	0.04 cd	0.01 ab	0.02 a	23.21 ab	38.16 abc	14.82
OH	ECB	10	0.38 a	0.18 a	4.42 abc	0.33 acdef	1.31	0.03 a	0.01 ab	0.01 a	22.29 abc	39.72 abc	16.42
OK	MDS	1	0.53 ab	0.21 ab	3.80 abcde	0.34 abcdef	1.29	0.03 abcd	0.01 ab	0.02 ab	24.64 abc	36.3 abc	13.70
PA	EC	1	0.15 ab	0.14 ab	4.61 abcde	0.47 abcdef	1.47	0.04 abcd	0.02 ab	0.02 ab	22.88 abc	39.55 abc	16.8
SD	WCB	18	0.41 a	0.18 a	4.64 ab	0.31 acf	1.36	0.03 a	0.01 b	0.01 a	22.32 abc	39.12 abc	15.46
TN	MDS	1	0.47 ab	0.24 ab	4.17 abcde	0.56 abcde	1.42	0.05 abcd	0.02 ab	0.02 ab	23.33 abc	37.25 abc	13.70
WI	WCB	5	0.30 a	0.13 a	5.51 ab	0.3 abcdef	1.47	0.03 abcd	0.01 ab	0.01 a	22.03 abc	39.88 abc	18.46

Average values are followed with group letters determined with the Tukey–Kramer HSD test. The Tukey–Kramer HSD test was employed to account for the difference in sample size between each group. Components with no significant differences between groups do not have letters. The East Corn Belt includes the states of IN, OH, WI, IL, and MI. The West Corn Belt includes the states of IA, MN, NE, ND, SD, and KS. The Mid-South includes the states of LA, AR, MS, and KY. The East Coast includes the state of PA. The results in this table are from samples obtained in 2020.

**Table 4:** Difference in traits of soybeans by region.

Region	*n*	Glucose	Fructose	Sucrose	Raffinose	Stachyose	Arabinose	Xylose	Melibiose	Oil	Protein	Seed Size
		(g 100 g^−1^)										(g 100 seeds ^−1^)
		2020 samples										
ECB	53	0.39 b	0.17 b	4.49 a	0.32 b	1.33	0.04 b	0.01	0.01 b	22.64	39.08 ab	16.08
EC	2	0.39 ab	0.22 ab	4.65 a	0.41 ab	1.42	0.04 ab	0.02	0.02 ab	22.17	39.77 ab	16.80
MDS	30	0.60 a	0.39 a	3.12 b	0.42 a	1.30	0.05 a	0.01	0.03 a	23.24	40.24 a	15.79
WCB	118	0.40 b	0.18 b	4.77 a	0.33 b	1.36	0.03 b	0.01	0.02 b	22.46	38.49 b	15.31
2021 samples												
ECB	11	NA	NA	NA	NA	NA	NA	NA	NA	20.71 a	42.15 b	17.65 b
WCB	9	NA	NA	NA	NA	NA	NA	NA	NA	21.60 a	37.50 a	16.27 ab
EC	1	NA	NA	NA	NA	NA	NA	NA	NA	23.48 ab	40.34 ab	16.80 ab
SE	2	NA	NA	NA	NA	NA	NA	NA	NA	24.11 ab	43.83 b	13.50 a
MDS	32	NA	NA	NA	NA	NA	NA	NA	NA	23.65 b	42.93 b	15.20 a

Average values are followed with group letters determined with the Tukey–Kramer HSD test. The Tukey–Kramer HSD test was employed to account for the difference in sample size between each group. Components with no significant differences between groups do not have letters. The East Corn Belt includes the states of IN, OH, WI, IL, and MI. The West Corn Belt includes the states of IA, MN, NE, ND, SD, and KS. The Mid-South includes the states of LA, AR, MS, and KY. The East Coast includes the state of PA. The Southeast includes the state of North Carolina.

## Data Availability

The original contributions presented in this study are included in the article/Supplementary Materials; further inquiries can be directed to the corresponding author.

## Supplementary Materials

The following supporting information can be downloaded at: https://www.mdpi.com/article/10.3390/foods13121884/s1, Table S1: Average protein and oil content and seed size of soybeans; Table S2: Seed size of 2021 samples by state; Table S3: Pearson correlation of soybean seed samples from 2020; Table S4: Pearson correlation of soybean seed samples from 2021.
