# Supplementary material for: Regional Variability in Sugar and Amino Acid Content of U.S. Soybeans and the Impact of Autoclaving on Reducing Sugars and Free Lysine
Source: Foods. 2024 Jun 15;13(12):1884. doi: 10.3390/foods13121884 (PMC11202694; doi:10.3390/foods13121884)
Supplement: Supplementary file 1 [file foods-13-01884-s001.zip › foods-3034877-supplementary.pdf]

Supplemental material

Table S1. Average Protein and Oil content and Seed Size of Soybeans

	Parameter	Average	Parameter	Average
Year	2020		2021	
	Oil (g 100^{-1}g)	22.60± 1.55	Oil (g 100^{-1g})	21.90 ± 2.02
	Protein (g 100^{-1}g)	38.91 ± 2.14	Protein (g 100^{-1g})	39.60 ± 3.63
	Seed size (g 100 seeds^{-1})	15.87 ± 2.57	Seed size (g 100 seeds^{-1})	16.28 ± 2.13

Average values are followed with ± standard deviation.

Table S2. Seed size of 2021 samples by state

**State**	**n**	**Seed size**	**State**	**n**	**Seed size**
AR	1	14.00ab	MS	1	15.40ab
IA	9	16.24ab	NC	2	13.50a
IL	3	16.43ab	ND	11	15.96a
IN	1	15.50ab	NE	2	17.20ab
KS	3	14.97a	OH	4	20.25b
KY	4	14.70a	PA	1	16.80ab
LA	3	16.20ab	SD	1	16.28ab
MI	1	17.60ab	WI	2	15.35ab
MN	6	17.20ab			

Average values are followed with group letters determined with the Tukey-Kramer HSD test.

Table S3. Pearson Correlation of Soybean Seed Samples from 2020

	Glucose	Fructose	Raffinose	Stachyose	Arabinose	Sucrose	Melibiose	Xylose	Oil	Seed_size	Protein
Glucose	1.00	0.69	0.17	0.17	0.43	-0.30	0.59	0.27	-0.06	-0.07	0.27
Fructose	0.69	1.00	0.17	0.15	0.49	-0.38	0.70	0.49	0.05	-0.08	0.27
Raffinose	0.17	0.17	1.00	0.21	0.49	-0.25	0.17	0.16	0.14	-0.09	0.16
Stachyose	0.17	0.15	0.21	1.00	0.26	0.49	0.14	0.14	-0.10	0.01	0.00
Arabinose	0.43	0.49	0.49	0.26	1.00	-0.29	0.44	0.56	0.04	-0.22	0.32
Sucrose	-0.30	-0.38	-0.25	0.49	-0.29	1.00	-0.33	-0.23	-0.14	0.16	-0.41
Melibiose	0.59	0.70	0.17	0.14	0.44	-0.33	1.00	0.45	0.04	-0.12	0.17
Xylose	0.27	0.49	0.16	0.14	0.56	-0.23	0.45	1.00	0.13	-0.18	0.10
Oil	-0.06	0.05	0.14	-0.10	0.04	-0.14	0.04	0.13	1.00	0.10	-0.46
Seed_size	-0.07	-0.08	-0.09	0.01	-0.22	0.16	-0.12	-0.18	0.10	1.00	0.11
Protein	0.27	0.27	0.16	0.00	0.32	-0.41	0.17	0.10	-0.46	0.11	1.00

Table S4. Pearson Correlation of Soybean Seed Samples from 2021

	Seed size	Protein	Oil	Arg	Lys	Ala	Thr	Gly	Val	Ser	Pro	Ile	Leu	Met	His	Phe	Glu	Asp	Cys	Tyr	Free lys	Glc	Fru	Suc	Lys loss	Glucose loss	Fructose loss	Sucrose loss
Seed size	1.00	0.08	-0.15	0.08	0.31	0.18	0.24	0.38	0.10	0.25	0.15	0.18	0.20	0.26	-0.01	0.24	0.04	0.11	0.27	0.21	-0.11	-0.13	-0.12	-0.10	0.16	0.10	-0.01	-0.10
Protein	0.08	1.00	-0.58	0.17	0.32	0.28	0.30	0.34	0.13	0.36	0.31	0.18	0.34	0.17	0.40	0.35	0.23	0.24	0.29	0.24	-0.14	-0.06	0.10	-0.13	-0.03	0.00	-0.06	0.05
Oil	-0.15	-0.58	1.00	0.02	-0.04	-0.08	-0.07	-0.12	0.09	-0.11	-0.09	0.07	-0.08	-0.04	-0.11	-0.06	-0.17	-0.15	-0.17	-0.15	0.14	0.16	0.01	0.11	0.00	-0.16	-0.03	-0.05
Arg	0.08	0.17	0.02	1.00	0.81	0.80	0.67	0.71	0.70	0.74	0.60	0.58	0.76	0.50	0.61	0.80	0.20	0.39	0.49	0.69	0.08	0.12	0.05	0.00	0.02	-0.08	-0.08	-0.03
Lys	0.31	0.32	-0.04	0.81	1.00	0.90	0.87	0.82	0.76	0.93	0.74	0.67	0.88	0.68	0.48	0.86	0.20	0.39	0.69	0.63	0.01	0.10	-0.01	-0.01	0.00	-0.09	-0.10	-0.08
Ala	0.18	0.28	-0.08	0.80	0.90	1.00	0.88	0.83	0.81	0.89	0.78	0.69	0.93	0.71	0.41	0.87	0.44	0.59	0.75	0.78	0.02	0.11	0.04	-0.05	-0.06	-0.01	0.04	-0.12
Thr	0.24	0.30	-0.07	0.67	0.87	0.88	1.00	0.82	0.78	0.93	0.88	0.79	0.92	0.81	0.27	0.83	0.43	0.57	0.88	0.66	-0.03	-0.04	-0.03	-0.09	-0.08	-0.05	-0.03	-0.11
Gly	0.38	0.34	-0.12	0.71	0.82	0.83	0.82	1.00	0.59	0.78	0.84	0.78	0.90	0.74	0.46	0.91	0.54	0.69	0.78	0.80	-0.06	0.01	0.05	-0.12	0.11	-0.06	-0.04	-0.10
Val	0.10	0.13	0.09	0.70	0.76	0.81	0.78	0.59	1.00	0.76	0.66	0.60	0.77	0.64	0.24	0.67	0.28	0.40	0.59	0.59	0.07	0.08	0.00	0.04	-0.05	0.01	0.11	-0.03
Ser	0.25	0.36	-0.11	0.74	0.93	0.89	0.93	0.78	0.76	1.00	0.76	0.69	0.90	0.69	0.41	0.83	0.32	0.46	0.77	0.63	0.00	0.01	-0.03	-0.13	-0.10	-0.02	-0.03	-0.16
Pro	0.15	0.31	-0.09	0.60	0.74	0.78	0.88	0.84	0.66	0.76	1.00	0.72	0.89	0.78	0.34	0.86	0.65	0.78	0.83	0.76	0.00	-0.04	-0.02	-0.16	0.07	0.00	0.05	0.04
Ile	0.18	0.18	0.07	0.58	0.67	0.69	0.79	0.78	0.60	0.69	0.72	1.00	0.81	0.74	0.31	0.75	0.42	0.54	0.70	0.64	-0.08	-0.06	0.14	-0.25	-0.03	-0.12	-0.13	-0.07
Leu	0.20	0.34	-0.08	0.76	0.88	0.93	0.92	0.90	0.77	0.90	0.89	0.81	1.00	0.74	0.47	0.94	0.55	0.69	0.80	0.82	0.03	0.08	0.06	-0.19	-0.01	-0.06	-0.04	-0.09
Met	0.26	0.17	-0.04	0.50	0.68	0.71	0.81	0.74	0.64	0.69	0.78	0.74	0.74	1.00	0.06	0.69	0.40	0.51	0.76	0.58	-0.02	-0.06	-0.04	-0.01	0.03	0.07	0.18	-0.01
His	-0.01	0.40	-0.11	0.61	0.48	0.41	0.27	0.46	0.24	0.41	0.34	0.31	0.47	0.06	1.00	0.63	0.12	0.23	0.10	0.47	-0.05	0.17	0.11	-0.24	0.02	-0.21	-0.21	0.03
Phe	0.24	0.35	-0.06	0.80	0.86	0.87	0.83	0.91	0.67	0.83	0.86	0.75	0.94	0.69	0.63	1.00	0.51	0.67	0.71	0.84	0.02	0.05	0.02	-0.20	0.03	-0.08	-0.06	-0.06
Glu	0.04	0.23	-0.17	0.20	0.20	0.44	0.43	0.54	0.28	0.32	0.65	0.42	0.55	0.40	0.12	0.51	1.00	0.94	0.63	0.74	-0.14	-0.09	0.01	-0.31	-0.07	0.02	0.07	-0.13
Asp	0.11	0.24	-0.15	0.39	0.39	0.59	0.57	0.69	0.40	0.46	0.78	0.54	0.69	0.51	0.23	0.67	0.94	1.00	0.74	0.87	-0.10	-0.10	-0.01	-0.26	-0.07	0.01	-0.01	-0.15
Cys	0.27	0.29	-0.17	0.49	0.69	0.75	0.88	0.78	0.59	0.77	0.83	0.70	0.80	0.76	0.10	0.71	0.63	0.74	1.00	0.73	-0.12	-0.18	-0.03	-0.09	-0.10	0.01	-0.02	-0.13
Tyr	0.21	0.24	-0.15	0.69	0.63	0.78	0.66	0.80	0.59	0.63	0.76	0.64	0.82	0.58	0.47	0.84	0.74	0.87	0.73	1.00	-0.07	-0.08	0.02	-0.23	-0.04	0.01	-0.04	-0.12
Free_lys	-0.11	-0.14	0.14	0.08	0.01	0.02	-0.03	-0.06	0.07	0.00	0.00	-0.08	0.03	-0.02	-0.05	0.02	-0.14	-0.10	-0.12	-0.07	1.00	0.22	0.00	0.15	0.46	0.07	0.11	0.16
Glc	-0.13	-0.06	0.16	0.12	0.10	0.11	-0.04	0.01	0.08	0.01	-0.04	-0.06	0.08	-0.06	0.17	0.05	-0.09	-0.10	-0.18	-0.08	0.22	1.00	0.19	0.05	0.03	-0.12	-0.03	-0.07
Fru	-0.12	0.10	0.01	0.05	-0.01	0.04	-0.03	0.05	0.00	-0.03	-0.02	0.14	0.06	-0.04	0.11	0.02	0.01	-0.01	-0.03	0.02	0.00	0.19	1.00	0.07	0.16	0.01	0.08	0.30
Suc	-0.10	-0.13	0.11	0.00	-0.01	-0.05	-0.09	-0.12	0.04	-0.13	-0.16	-0.25	-0.19	-0.01	-0.24	-0.20	-0.31	-0.26	-0.09	-0.23	0.15	0.05	0.07	1.00	-0.03	0.02	0.01	0.10
Lys loss	0.16	-0.03	0.00	0.02	0.00	-0.06	-0.08	0.11	-0.05	-0.10	0.07	-0.03	-0.01	0.03	0.02	0.03	-0.07	-0.07	-0.10	-0.04	0.46	0.03	0.16	-0.03	1.00	0.15	0.28	0.56
Glucose loss	0.10	0.00	-0.16	-0.08	-0.09	-0.01	-0.05	-0.06	0.01	-0.02	0.00	-0.12	-0.06	0.07	-0.21	-0.08	0.02	0.01	0.01	0.01	0.07	-0.12	0.01	0.02	0.15	1.00	0.55	0.10

Fructose loss	-0.01	-0.06	-0.03	-0.08	-0.10	0.04	-0.03	-0.04	0.11	-0.03	0.05	-0.13	-0.04	0.18	-0.21	-0.06	0.07	-0.01	-0.02	-0.04	0.11	-0.03	0.08	0.01	0.28	0.55	1.00	0.24
Sucrose loss	-0.10	0.05	-0.05	-0.03	-0.08	-0.12	-0.11	-0.10	-0.03	-0.16	0.04	-0.07	-0.09	-0.01	0.03	-0.06	-0.13	-0.15	-0.13	-0.12	0.16	-0.07	0.30	0.10	0.56	0.10	0.24	1.00

Abbreviations; Glc: glucose, Fru: fructose, Suc: Sucrose.
